# Suppression of interferon gene expression overcomes resistance to MEK inhibition in *KRAS*-mutant colorectal cancer

**DOI:** 10.1038/s41388-018-0554-z

**Published:** 2018-10-23

**Authors:** Steve Wagner, Georgios Vlachogiannis, Alexis De Haven Brandon, Melanie Valenti, Gary Box, Liam Jenkins, Caterina Mancusi, Annette Self, Floriana Manodoro, Ioannis Assiotis, Penny Robinson, Ritika Chauhan, Alistair G. Rust, Nik Matthews, Kate Eason, Khurum Khan, Naureen Starling, David Cunningham, Anguraj Sadanandam, Clare M. Isacke, Vladimir Kirkin, Nicola Valeri, Steven R. Whittaker

**Affiliations:** 1 Division of Cancer Therapeutics, The Institute of Cancer Research, London, UK; 2 Division of Molecular Pathology, The Institute of Cancer Research, London, UK; 3 Breast Cancer Now Research Centre, The Institute of Cancer Research, London, UK; 4 Tumour Profiling Unit, The Institute of Cancer Research, London, UK; 5 Department of Medicine, Royal Marsden NHS Foundation Trust, London, UK

**Keywords:** Colorectal cancer, Cancer genomics

## Abstract

Despite showing clinical activity in *BRAF*-mutant melanoma, the MEK inhibitor (MEKi) trametinib has failed to show clinical benefit in *KRAS*-mutant colorectal cancer. To identify mechanisms of resistance to MEKi, we employed a pharmacogenomic analysis of MEKi-sensitive versus MEKi-resistant colorectal cancer cell lines. Strikingly, interferon- and inflammatory-related gene sets were enriched in cell lines exhibiting intrinsic and acquired resistance to MEK inhibition. The bromodomain inhibitor JQ1 suppressed interferon-stimulated gene (ISG) expression and in combination with MEK inhibitors displayed synergistic effects and induced apoptosis in MEKi-resistant colorectal cancer cell lines. ISG expression was confirmed in patient-derived organoid models, which displayed resistance to trametinib and were resensitized by JQ1 co-treatment. In in vivo models of colorectal cancer, combination treatment significantly suppressed tumor growth. Our findings provide a novel explanation for the limited response to MEK inhibitors in *KRAS*-mutant colorectal cancer, known for its inflammatory nature. Moreover, the high expression of ISGs was associated with significantly reduced survival of colorectal cancer patients. Excitingly, we have identified novel therapeutic opportunities to overcome intrinsic and acquired resistance to MEK inhibition in colorectal cancer.

## Introduction

Common genetic alterations responsible for the development and progression of colorectal cancer (CRC) include inactivation of the tumor suppressors *APC* and *TP53* and mutational activation of *KRAS* [1, 2]. A recently described model of inducible *Apc* and *Trp53* loss and *Kras*^*G12D*^ expression in colonic intestinal epithelial cells demonstrated this by recapitulating the progression from adenoma to carcinoma, with a key role of *Kras*^*G12D*^ being to accelerate tumorigenesis and increase the incidence of metastatic disease [3]. Importantly, extinction of *Kras*^*G12D*^ in tumors caused them to revert to adenomas, underscoring their continued dependence on mutant Kras and providing further confirmation that Kras signaling remains an important driver of late-stage disease. Increasing evidence implicates oncogenic Ras in the modulation of the tumor microenvironment to support tumor growth [4, 5]. This is achieved by paracrine signaling from tumor cells to the stroma via secretion of cytokines, such as interleukin-6 (IL-6) and IL-8 (CXCL8), which promote invasion, neovascularization, and inflammatory responses [6, 7]. Notably, genetic or pharmacological approaches to target cytokines or their receptors have shown promising signs of antitumor activity [6, 8, 9]. However, there remain concerns that targeting individual cytokines or their receptors may be insufficient and that broader blockade of cytokine networks may be required for therapeutic efficacy.

Current approved targeted therapies for CRC include anti-angiogenic drugs, such as bevacizumab and regorafenib, as well as epidermal growth factor receptor inhibitors cetuximab and panitumumab for *KRAS* wild-type cancer [10–13]. The demonstration that oncogenic KRAS prompted activation of the mitogen-activated protein kinase (MAPK) pathway prompted concerted efforts to develop inhibitors of mitogen-activated protein kinase kinase (MEK), a key intermediary of KRAS signaling [14]. This work culminated in the Food and Drug Administration approval of the MEK inhibitor (MEKi) trametinib for *BRAF*-mutant melanoma [15]. However, trametinib failed to demonstrate significant clinical activity in other *RAS*-mutant cancers, including CRC [16].

Resistance to MEKis has been attributed to mutation of the drug-binding site of MEK [17], or through suppression of negative feedback regulation of receptor tyrosine kinases such as ERBB3 and FGFR1 [18, 19] and Raf-1 proto-oncogene, serine/threonine kinase (CRAF)-mediated reactivation of MEK [20]. Our study has focussed on identifying pre-existing transcriptional states associated with resistance that may not have been elucidated by the kinome-focussed, RNA interference screens used in prior studies [18–20]. We hypothesized that cell lines exhibiting intrinsic resistance to MEK inhibition may have distinct transcriptional profiles, which render them indifferent to MAPK pathway inhibition. To this end, we utilized a pharmacogenomics analysis of *KRAS*-mutant CRC cell lines with differing sensitivity to pharmacologic MEK inhibition and identified transcriptional states associated with resistance. We demonstrate a striking enrichment of interferon- and inflammation-regulated genes in MEKi-resistant cell lines and importantly, we further associate these transcriptional states to the development of acquired resistance to MEK inhibition. Moreover, we describe in colorectal cell lines, organoids from metastatic patient samples and in xenograft and syngeneic models, a therapeutic strategy to suppress inflammatory gene expression, restore sensitivity to MEK inhibition and forestall the emergence of drug-resistant populations.

## Results

### Elevated expression of inflammatory/interferon-stimulated genes (ISGs) is associated with resistance to MEK inhibition

**Fig. 1:**
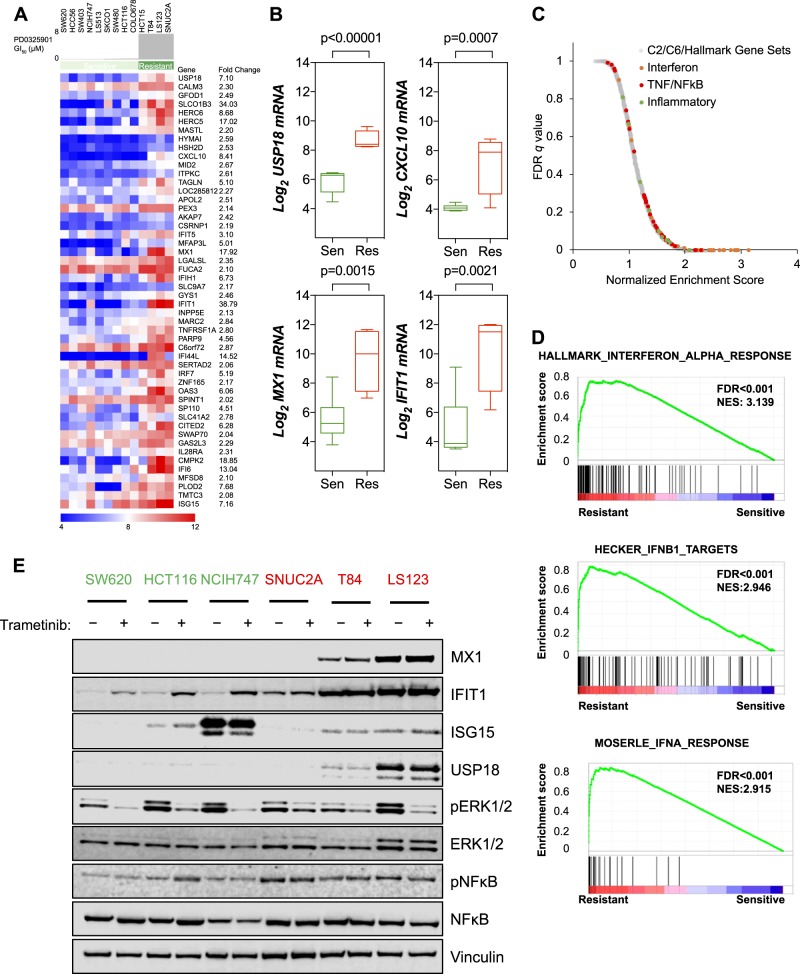
Multiple inflammatory gene expression signatures are enriched in MEK inhibitor-resistant colorectal cancer cell lines. **a** Differential expression analysis (comparative marker selection, Morpheus, The Broad Institute) of basal gene expression profiles for *KRAS*-mutant colorectal cancer cell lines identified genes that were differentially overexpressed in cells resistant to the MEK inhibitor PD0325901 (top 50 genes shown). **b** Box and whisker plots representing the expression of candidate resistance genes in MEK inhibitor-sensitive versus MEK inhibitor-resistant cell lines. Box indicates the 25–75% percentiles and whiskers are the minimum to maximum values. **c** Gene set enrichment analysis (GSEA) of the rank-ordered, differentially expressed genes in MEK inhibitor-resistant cell lines identified an enrichment of multiple inflammation-related gene sets. **d** GSEA identified interferon response genes to be significantly enriched in the resistant cell lines (FDR < 0.001, *p* < 0.001). **e** Cells were treated with DMSO or 30 nmol/L trametinib for 3 d and cell lysates analyzed by western blotting for the indicated proteins

We set out to identify gene expression differences between *KRAS*-mutant, CRC cell lines that were either sensitive or resistant to MEK inhibition. Utilizing the Cancer Cell Line Encyclopedia (CCLE), we classified the 13 cell lines based on their GI_50_ to the second-generation MEKi PD0325901 [21]. Four cell lines were classified resistant (GI_50_ > 8 µmol/L) and nine were classified as sensitive (GI_50_ < 250 nmol/L). We used comparative marker selection to identify genes that were differentially expressed between the two groups and focussed on the 140 genes that showed increased expression in the resistant cell lines by a factor of two fold or greater (Fig. 1a). We confirmed that the mRNA expression of *USP18*, *CXCL10*, *MX1* and *IFIT1* was significantly increased in resistant cell lines (Fig. 1b). Unbiased gene set enrichment analysis (GSEA) demonstrated that interferon- and inflammation-related gene sets were enriched in the resistant cells (Fig. 1c) and the three top-ranking gene sets were characteristic of responses to interferon alpha and beta (Fig. 1d).

Recently, the MEKi trametinib was approved for the treatment of *BRAF*-mutant melanoma. However, trametinib failed to show any activity in *BRAF* or *KRAS*-mutant CRC [16]. Based on our data above and the findings that inflammation can drive the development of CRC, that oncogenic KRAS is known to induce an inflammatory environment in the colon, and that chemotherapies also cause increased inflammation in the colon, we hypothesized that intrinsic or chemotherapy-induced inflammation may result in a tumor microenvironment that renders cells resistant to trametinib [22–24]. Therefore, we first confirmed that cell lines known to be resistant to PD0325901 also displayed resistance to trametinib (GI_50_ > 10 nmol/L) (Figure S1). We assessed the expression of some of the genes identified above at the protein level and found that IFIT1, MX1, and USP18 were more abundant in MEKi-resistant cell lines T84 and LS123, whereas ISG15 showed more variable expression (Fig. 1e, Fig S2). SNUC2A cells did not express MX1 or USP18 but did show greater expression of IFIT1 compared with untreated, sensitive cell lines. In the resistant T84 and LS123 cell lines, treatment with trametinib had little effect on the (already high) expression of MX1, IFIT1, and USP18, but induced the expression of IFIT1 in the sensitive cell lines and in the SNUC2A cells (Fig. 1e). We also observed a trend for higher levels of nuclear factor kappa B (NFκB) phosphorylation in the MEKi-resistant cell lines. Consistent with our data, we found evidence for increased expression of various ISGs in MEKi-resistant T84 and LS123 cells in a recently published proteomics dataset [25] (Figure S3A). GSEA analysis of the proteomics data confirmed significant enrichment of interferon gene sets in the resistant cell lines (Figure S3B). Overall, these data suggested that increased ISG expression is not only associated with intrinsic resistance to MEK inhibition but can be induced by treatment in sensitive cell lines.

### Acquired resistance to MEK inhibition results in ISG expression and subtype switching

**Fig. 2:**
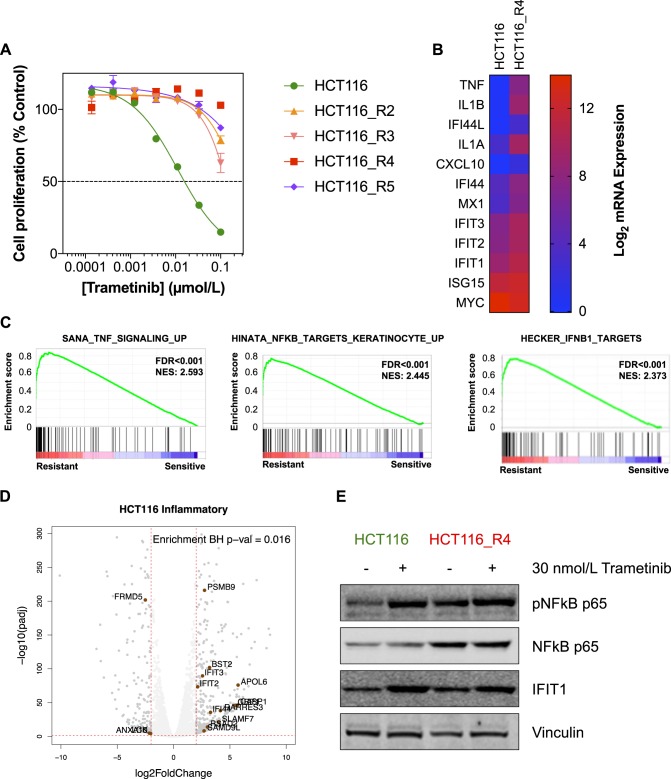
Acquired resistance to trametinib is associated with inflammatory gene expression and NFκB activation. **a** Trametinib-resistant HCT116 subclones were derived through chronic exposure to the compound over 4–8 weeks. These clones demonstrated a > 10-fold increase in the GI_50_ for trametinib compared with the parental control. Mean cell proliferation values shown as a percentage of control cells is plotted, with error bars representing standard error (*n* = 3). **b** RNA-seq of the HCT116 and HCT116_R4 cell lines was used to profile transcriptional changes in the trametinib-resistant clone. Increased expression of various inflammatory genes identified in Fig. 1a was observed (mean log_2_ values shown, *n* = 3 replicates per condition). **c** GSEA of RNA-seq data identified an enrichment of inflammatory gene signatures in the HCT116_R4 clone, with TNFα and NFκB gene sets being the most highly ranked (FDR < 0.001, *p* < 0.001). **d** Significant enrichment of genes associated with the inflammatory molecular subtype, indicates potential change of the parental HCT116 stem-like subtype to the inflammatory subtype with an increased set of inflammatory-specific genes. **e** HCT116 and HCT116_R4 cells were treated with 30 nmol/L trametinib for 72 h and lysates were analyzed by western blotting for the indicated proteins

Given that IFIT1 expression was induced in sensitive cell lines following 72-h treatment with trametinib, we hypothesized that an adaptive response to MEK inhibition would be to upregulate ISGs and this might contribute toward acquired resistance to trametinib. Therefore, we treated HCT116 human colon cancer cells with increasing concentrations of trametinib over 2 months. Drug-resistant clones emerged and were cultured in the presence of 30 nmol/L trametinib. These cells exhibited a >10-fold increase in the GI_50_ for trametinib compared with the parental cell line (Fig. 2a). RNA-seq of the resistant clone HCT116_R4 versus the parental cells identified many of the ISGs that we previously identified to be overexpressed in the intrinsically resistant cell lines (Fig. 2b). We confirmed increased expression of some of these immune-related genes, including tumor necrosis factor-α (TNFα) and IL1α by reverse transcriptase-quantitative PCR (RT-qPCR) in additional, trametinib-resistant clones (Figure S4A). Moreover, addition of recombinant TNFα, or IL1α to the culture medium of HCT116 cells was sufficient to confer resistance to trametinib (Figure S4B), alongside activation of NFκB (Figure S4C). GSEA of the RNA-seq data revealed that inflammatory/interferon-related gene sets including TNFα signaling, NFκB target genes, and interferon response genes were ranked in the top 6 gene sets (Fig. 2c, Table S1). Furthermore, a significant enrichment of inflammatory marker genes that signify the inflammatory subtype of CRC was present in the HCT116_R4 cell line (Fig. 2d). This suggests that the trametinib-resistant HCT116 colon cancer cells may have transitioned from the stem-like subtype to the inflammatory subtype, as defined by Sadanadam et al. [26]. Given the increase in NFκB target genes, as highlighted by the RNA-seq data, the activation state of NFκB was verified by western blotting. In the parental HCT116 cell line, treatment with trametinib-induced NFκB p65 phosphorylation and increased the expression of IFIT1. In the resistant HCT116_R4 cells, basal NFκB phosphorylation and expression was notably higher, relative to the parental cells, and basal IFIT1 expression was also elevated (Fig. 2e, Figure S5). Altered expression of USP18 and MX1 was not detected (data not shown). Taken together, these data support our hypothesis that an interferon/inflammatory gene expression program operates both in intrinsically MEKi-resistant colon cancer cells and in those that acquire resistance to trametinib.

### Inhibition of bromodomain proteins suppresses inflammatory gene expression and restores sensitivity to trametinib

**Fig. 3:**
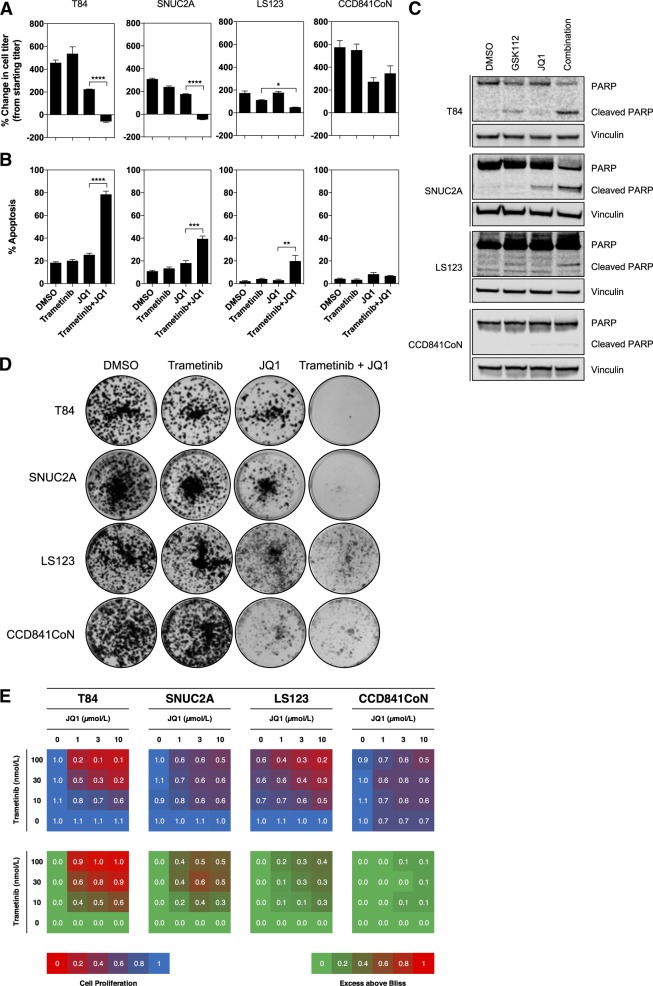
Synchronous inhibition of MEK and bromodomain-containing proteins inhibits cell proliferation and induces cell death in colon cancer cell lines. **a** MEK inhibitor-resistant human colon cell lines, T84, SNUC2A, and LS123 or the normal colon epithelial cell line CCD841CoN were treated with 30 nmol/L trametinib or 1 µmol/L JQ1 for 72 h. Cell proliferation was determined by cell counting and expressed as a percentage of the cell number prior to treatment. Mean values are presented, ± standard error (*n* = 3). Statistical significance was determined using a one-way ANOVA *p < 0.05, **p < 0.01, ***p < 0.001, ****p < 0.0001. **b** Cells were treated as in (**a**) and then analyzed for annexin V positivity by flow cytometry. The mean percentage of annexin V-positive cells relative to DMSO controls is shown, ± standard error (*n* = 3). **c** Cells were treated as in (**a**) and cell lysates were analyzed by western blotting for the indicated proteins. **d** T84, SNUC2A, and LS123 cells, or the normal colon epithelial cell line CCD841CoN were treated with 30 nmol/L trametinib, 1 µmol/L JQ1 or the combination of both compounds for 14 d and cell proliferation was assessed by colony formation assay. **e** T84, SNUC2A, and LS123 cells, or the normal colon epithelial cell line CCD841CoN were treated with a matrix of trametinib and JQ1 for 4 d, and cell proliferation was assessed by the CellTiter-Blue assay (decrease in proliferation is shown by a shift from blue to red). Synergy was determined by the Bliss independence model (the excess above bliss score is indicated, with red indicating synergy)

Given that inflammatory gene expression appeared to associate with resistance to MEK inhibition, we hypothesized that its suppression may restore sensitivity to trametinib in resistant cell lines. The bromodomain inhibitor JQ1 inhibits inflammatory gene expression by the suppression of inflammatory gene super enhancers and via inhibition of NFκB p65 (*RELA*) and NFκB-driven super enhancers [27, 28]. Therefore, we tested the effect of combined trametinib and JQ1 treatment on MEKi-resistant cell lines. Treatment of T84, SNUC2A, and LS123 cells with either trametinib or JQ1 alone had only modest effects on cell proliferation, whereas the combination of both compounds resulted in a reduction of cell proliferation, including a net loss of cells relative to the number prior to treatment for T84 and SNUC2A cell lines (Fig. 3a). Notably, the proliferation rate of CCD841CoN colorectal normal epithelial cells was reduced by JQ1 alone and the combination of trametinib and JQ1 but not to the same extent as the cancer cell lines. A significantly increased apoptotic population was observed with the drug combination versus dimethylsulfoxide (DMSO) or single-agent treatment, as determined by annexin V staining (Fig. 3b) and poly(ADP-ribose) polymerase (PARP) cleavage (Fig. 3c). Only a modest increase in annexin V staining and PARP cleavage was observed in CCD841CoN cells, which appeared to be mainly in response to JQ1 treatment. In colony assays, trametinib and JQ1 had little effect on their own but their combination robustly inhibited proliferation of the cancer cell lines. However, in the CCD841CoN epithelial cells JQ1 treatment alone did significantly reduce cell proliferation and consequently no additional benefit of the combination was observed (Fig. 3d, Figure S6). We employed the Bliss independence model to assess the combination of trametinib and JQ1 and observed synergy across a matrix of concentrations for each agent (Fig. 3e). In agreement with the above, only slight synergy was observed in the CCD841CoN colon epithelial cells.

**Fig. 4:**
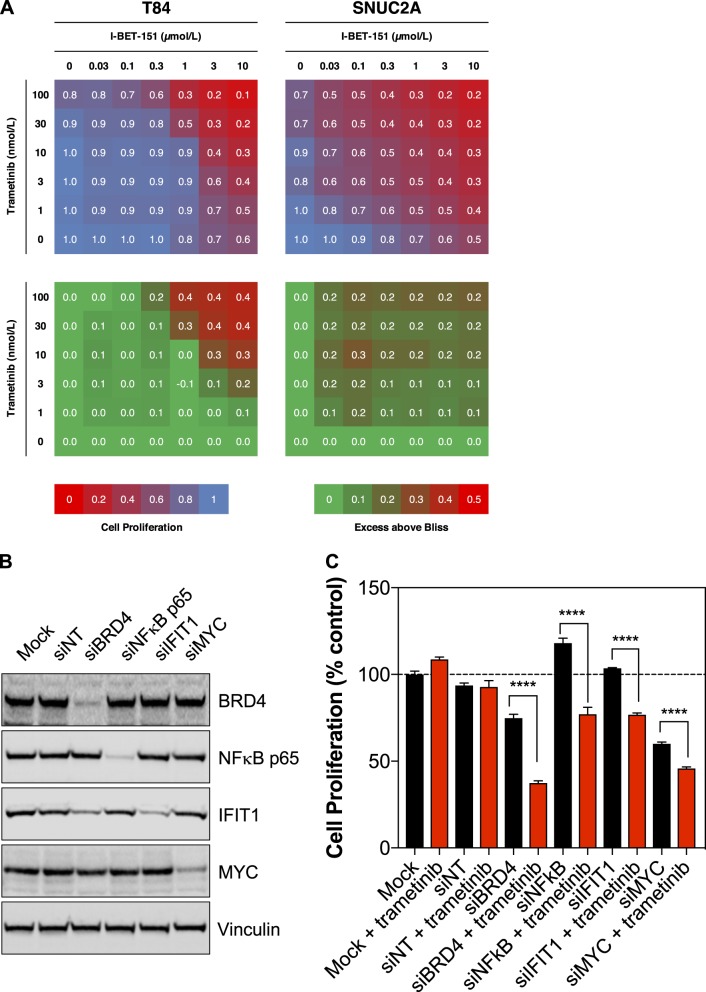
Inhibition of BRD4 via I-BET-151 or siRNA enhances sensitivity to trametinib. **a** T84 and SNUC2A cells were treated with a matrix of trametinib and I-BET-151 for 4 d and cell proliferation was quantified by the CellTiter-Blue assay. Inhibition of cell proliferation is indicated by a shift from blue to red, and synergy, as determined by the Bliss independence model, is indicated by a shift from green to red. **b** T84 cells were reverse-transfected with 100 nM of an siRNA Smart Pools targeting BRD4, NFκB, IFIT1, MYC or a non-targeting control for 7 d and cell lysates were analyzed by western blotting for the indicated proteins (*n* = 3). **c** Cells were treated as in (**b**) in the presence of DMSO or 30 nmol/L trametinib and cell proliferation was determined by the CellTiter-Blue assay. Mean values are presented, ± standard error (*n* = 6). Statistical significance was determined using a one-way ANOVA ****p < 0.0001

**Fig. 5:**
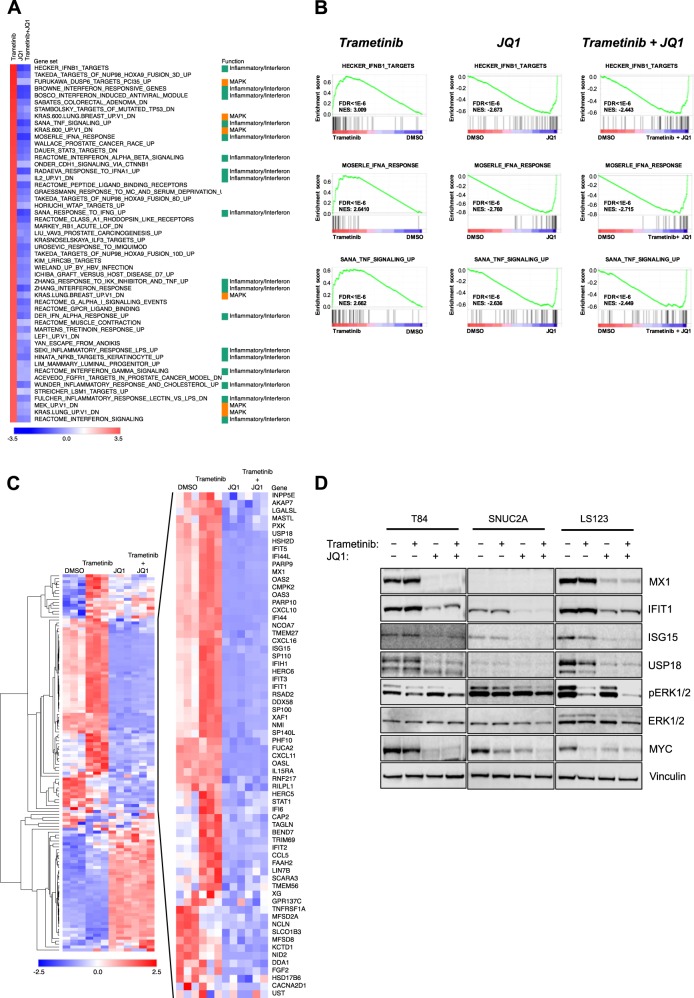
Inhibition of inflammatory gene expression by JQ1. **a** T84 cells were treated with DMSO, 30 nmol/L trametinib, 1 µmol/L JQ1, or their combination for 24 h and analyzed by RNA-seq in triplicate. GSEA showed the enrichment of inflammatory-related gene sets following trametinib exposure, and their subsequent depletion following treatment with JQ1 or the combination of JQ1 and trametinib. Gene sets are ordered by the normalized enrichment score for the trametinib-treated condition. Gene sets unaffected in the JQ1 or JQ1 and trametinib conditions were excluded from the data. **b** GSEA plots of specific gene sets enriched in MEK inhibitor-resistant cell lines, previously identified in the CCLE dataset. Enrichment is further enhanced by trametinib treatment; however, following treatment with JQ1, or JQ1 and trametinib combination, these genes sets are among the most significantly depleted gene sets. **c** Data for the 140 genes implicated in resistance to PD0325901 in the CCLE dataset were extracted from the RNA-seq analysis of T84 cells treated as in (**a**). Hierarchical clustering was used to group genes according to their pattern of expression across the different treatments. A cluster of 66 genes that was induced by trametinib and suppressed by JQ1 or JQ1 and trametinib combinatorial treatment was apparent. **d** T84, SNUC2A, and LS123 cells were treated with DMSO, 30 nmol/L trametinib, 1 µmol/L JQ1, or their combination for 72 h. Cell lysates were analyzed for the indicated proteins

Consistent with best practice for the use of chemical probes [29], we used a second, chemically distinct bromodomain inhibitor, I-BET-151, also shown to suppress inflammatory gene expression [30], and confirmed that it too could sensitize cells to trametinib (Fig. 4a). In addition, we used small interfering RNAs (siRNAs) against BRD4 or NFκB p65 to achieve robust decreases in BRD4 or NFκB p65 protein expression (Fig. 4b). Increased antiproliferative activity of trametinib was observed with the combination of BRD4 or NFκB knockdown (Fig. 4c). Furthermore, knockdown of IFIT1 or MYC, a known BRD4 target gene [31] (Fig. 4b), also sensitized the cells to trametinib (Fig. 4c). Notably, knockdown of BRD4 also led to a decrease in the expression of IFIT1 (but not MYC) providing further evidence for regulation of IFIT1 by BRD4 and suggesting it may be necessary to target multiple BET family proteins to suppress MYC expression (Fig. 4b). Therefore, genetic suppression of BRD4, NFκB, IFIT1, or MYC sensitizes cells to MEK inhibition, raising confidence that suppression of BRD4, NFκB, IFIT1, and MYC may contribute to the effect of JQ1. To investigate this at the level of transcriptional regulation, we performed RNA-seq of T84 cells treated for 24 h with DMSO, trametinib, JQ1, or the combination of trametinib and JQ1. Treatment with trametinib resulted in increased expression of inflammatory genes, with GSEA analysis again showing inflammation- and interferon-regulated gene sets to be highly enriched under these conditions (Figs. 5a, b). Treatment with JQ1, either alone or in the presence of trametinib resulted in a marked reduction of inflammatory/interferon-regulated genes with the gene sets we had previously associated with resistance being ranked as the most depleted (Figs. 5a, b). Furthermore, by examining the expression of the 140 genes initially identified in the CCLE dataset as being upregulated in MEKi-resistant cell lines, a cluster of JQ1-sensitive inflammatory/interferon genes emerged. These genes were mostly induced by trametinib treatment and were repressed by JQ1, either alone or in combination with trametinib (Fig. 5c, Table S2). We confirmed the suppression of inflammatory proteins by JQ1 in T84, SNUC2A, and LS123 colon cancer cells treated with trametinib, JQ1, or both agents combined for 72 h (Fig. 5d). The expression of MX1, IFIT1, ISG15, and MYC was reduced by JQ1, either alone or in combination with trametinib. The combination treatment also led to slightly greater inhibition of ERK1/2 phosphorylation compared with either agent alone.

We hypothesized that treatment with JQ1 would suppress the emergence of acquired resistance to trametinib. When cultured in the presence of 30 nmol/L trametinib, HCT116 cells initially responded but by 4 weeks of treatment cells became resistant to trametinib and formed viable colonies. Treatment with 300 nmol/L JQ1 alone had a modest effect on cell proliferation but the combination of both agents robustly suppressed the emergence of resistant colonies (Figure S7A). We further confirmed this in HCT116 cells grown as spheroids (Figure S7B). We next assessed whether cells that had acquired resistance to trametinib could be challenged successfully with JQ1 at a later point in time. Compared with the parental cell line, the trametinib-resistant clones were up to five-fold more sensitive to JQ1 (Figure S7C). Notably, compared with the parental line, the HCT116_R4 cell line was dramatically more susceptible to long-term treatment with JQ1, either in the presence or absence of trametinib, as shown by colony formation assay (Figure S7D). Notably, proliferation of the HCT116_R4 clone was impaired when trametinib was washed out, suggesting the cells had adapted to proliferate in the presence of trametinib. JQ1 treatment suppressed trametinib-induced IFIT1 expression in the HCT116 cells and reduced both basal and trametinib-induced IFIT1 expression in the HCT116_R4 cells (Figure S7E). Taken together, these data demonstrate that ISG expression is observed in cell lines exhibiting intrinsic or acquired resistance to trametinib and that suppression of ISG expression restores sensitivity to trametinib and suppresses the emergence of acquired resistance.

### Patient-derived CRC organoids express inflammatory genes, are resistant to trametinib but are sensitive to dual JQ1/trametinib treatment

**Fig. 6:**
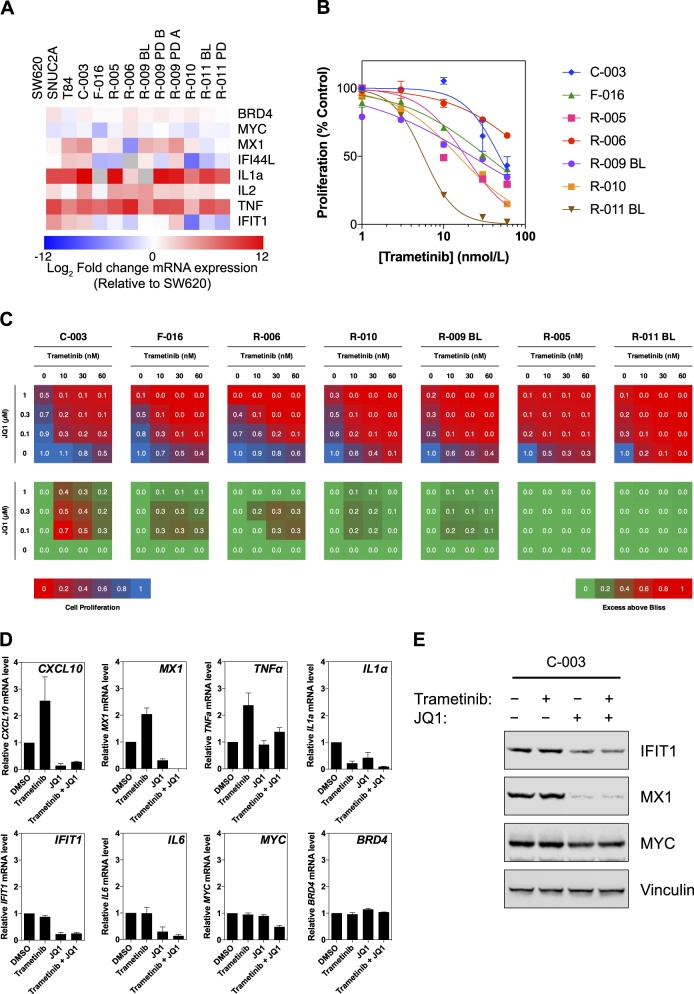
The combination of trametinib and JQ1 is efficacious in patient-derived organoid models of *KRAS*-mutant colorectal cancer. **a** Patient-derived organoid cultures generated from *KRAS*-mutant colorectal cancer biopsies were profiled for mRNA expression of the indicated genes by RT-qPCR. Values are expressed relative to the MEK inhibitor-sensitive SW620 cell line. **b** Organoid cultures were treated with a titration of trametinib for 7 d and proliferation was assessed by the CellTiter-Blue assay. Data are presented as percentage of DMSO-treated organoids (*n* = 3). **c** Seven different organoid cultures were treated with a matrix of trametinib and JQ1 for 7 d. Organoid proliferation was assessed as in (**b**); a shift from blue to red indicates reduced proliferation. Synergy was determined using the Bliss independence model; a shift from green to red indicates an excess above bliss, indicative of synergy (*n* = 3). **d** RT-qPCR was performed on the C-003 organoid culture treated with either DMSO, 10 nM trametinib, 100 nM JQ1, or their combination for 24 h for expression of the indicated genes. Mean values are relative to DMSO-treated control, normalized to *GUSB* expression; error bars represent standard error (*n* = 2–3). **e** The C-003 organoid culture was treated as described in (**d**) for 48 h and protein lysates were analyzed for the indicated proteins

To test the hypothesis that *KRAS*-mutant CRCs display high expression of inflammatory genes, which may predispose them to be resistant to MEK inhibition, we made use of a panel of patient-derived organoid (PDO) cultures from *KRAS*-mutant CRCs [32]. Compared with the trametinib-sensitive cell line SW620, and in common with the T84 and SNUC2A trametinib-resistant cell lines, the PDOs exhibited elevated expression of inflammatory genes such as *MX1*, *IFI44L*, *IL1*α, *IL2*, and *TNF*α (Fig. 6a). Furthermore, all but one of the PDO cultures (R-011 BL, which has a gain of BRAF) were classified as resistant to trametinib with GI_50_ values in excess of 10 nmol/L (Fig. 6b). Excitingly, in those PDOs that were most resistant to trametinib we found that sensitivity could be restored by co-treatment with JQ1 and that this combination was highly synergistic in 5/7 PDO cultures (Fig. 6c). Trametinib treatment increased *CXCL10*, *MX1*, and *TNF*α mRNA expression but their expression and that of *IL1*α, *IFIT1*, and *IL6* was reduced to basal levels or less by JQ1 treatment (Fig. 6d). Notably, the combination of trametinib and JQ1 did lead to more complete suppression of genes that reflect the resistant state, eg., MX1, IL1α, IL-6, and MYC expression. Inhibition of MX1, IFIT1, and MYC protein expression was observed with combined treatment (Fig. 6e, Figure S8). These data therefore provide key, clinically relevant support to our hypothesis that CRCs may be influenced by inflammatory environments or may engage inflammatory pathways or transcriptional programs that promote resistance to trametinib, and that the rational combination of bromodomain inhibitors and trametinib is a potential therapeutic strategy.

### The combination of trametinib and JQ1 suppresses the growth of *KRAS*-driven tumors in vivo

**Fig. 7:**
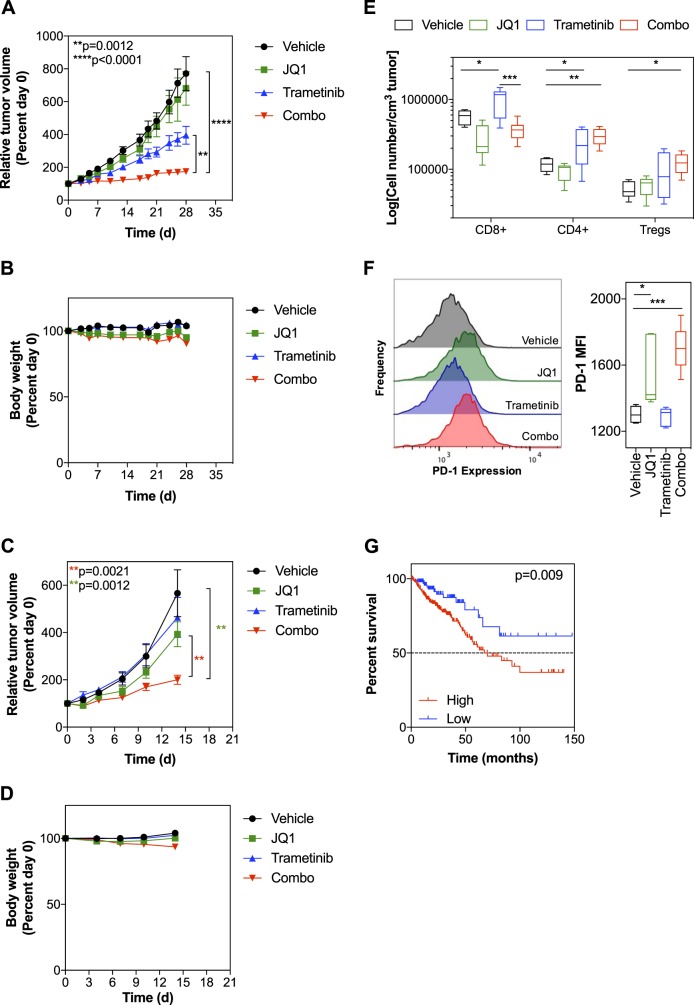
The combination of trametinib and JQ1 is efficacious in MEK inhibitor-resistant animal models. **a** Human T84 cells (5 × 10^6^/mouse) were inoculated subcutaneously into the flank of NCr mice, *n* = 10 mice per group. Mice were treated with either vehicle, 1 mg/kg/d po trametinib, 50 mg/kg/d ip JQ1, or the combination of trametinib and JQ1 for up to 28 d. Dosing on day 21 was withheld from all groups to aid tolerability. Tumor volume was measured by callipers every 3–5 d, and the mean volume per group was expressed as a percentage relative to day 0; error bars represent standard error. Statistical significance was determined using a two-tailed *t*-test of relative tumor volumes after 28 d of dosing. **b** The body weight of the mice from each group in (**a**) was measured and the mean per group was expressed as a percentage change from day 0; error bars represent standard error. **c** Mouse CT26 cells (5 × 10^5^/mouse) were inoculated subcutaneously into the flank of BALB/c mice. When tumors reached approximately 100 mm^3^ mice were treated with 1 mg/kg/d po trametinib, 50 mg/kg/d ip JQ1, or the combination of both compounds (*n* = 7-8 mice/group). Tumor volume was measured by callipers every 3–5 d, and the mean volume per group was expressed as a percentage relative to day 0; error bars represent standard error. Statistical significance was determined using a two-tailed *t*-test of relative tumor volumes after 14 d of dosing. **d** The body weight of the mice from each group in (**c**) was measured and the mean per group was expressed as a percentage change from day 0; error bars represent standard error. **e** Quantification of T-cell populations (CD8+, CD4+, Tregs) in CT26 tumors from (**c**), assessed by multi-color flow cytometry. Cell numbers are expressed as the number of cells per cm^3^ of tumor, presented in the box and whisker plot. Statistical significance was determined using a one-way ANOVA, *n* = 6 mice per group, *p < 0.05, **p < 0.01, ***p < 0.001. **f** The expression of PD-1 was determined by flow cytometry in CD8+ T cells isolated from CT26 tumors from (**c**). An example histogram is shown for each condition and aggregate data is presented in the box and whisker plot. Statistical significance was determined using a one-way ANOVA, *n* = 6 mice per group, *p < 0.05, **p < 0.01, ***p < 0.001. **g** Overall survival of 379 colorectal cancer patients with high expression (mRNA z-score > 2) of 66 genes identified in Fig. 5c (cBioportal). Significance was determined by log-rank (Mantel–Cox) test

We wished to confirm that our therapeutic approach of inhibiting bromodomain proteins to overcome resistance to MEK inhibition is tolerated and efficacious in animal models and established the *KRAS*-mutant, T84 cell line as a xenograft model of intrinsic resistance to MEK inhibition in NCr nude mice. Once tumors were established, we treated mice with vehicle, trametinib, JQ1 or the combination of trametinib and JQ1 (Fig. 7a). JQ1 alone had little effect on tumor growth, whereas trametinib slowed tumor growth by ~50%. However, the combination of both agents resulted in near-complete suppression of tumor growth during the 28-d dosing period. This dosing schedule was well tolerated and any weight loss was within acceptable limits (Fig. 7b). On termination of treatment, tumor growth resumed in the trametinib and combination groups (Figure S9A), with only the combination group significantly inhibiting tumor growth out to 42 d. The combination led to an improved median survival of 74.5 d, which approached significance (*p* = 0.0512), versus 52.5 d with trametinib alone, 42 d with JQ1 alone (both not significant) when compared with 44.5 d with vehicle (Figure S9B). The combination treatment gave a significantly improved survival compared with JQ1 treatment alone (*p* = 0.0131) but this was not significantly better than trametinib alone (*p* = 0.4357). To confirm the efficacy of this combination in an immunocompetent model, we used the *Kras*-mutant, CT26 mouse syngeneic model in BALB/C mice. Whereas trametinib and JQ1 failed to slow tumor growth, the combination of both agents markedly suppressed tumor growth over 14 d (Fig. 7c) and was well tolerated by the mice (Fig. 7d).

Given the potential of trametinib and JQ1 to alter the tumor immune cell landscape by modulating inflammatory gene expression (as described herein) or by direct effects on immune cells, we assessed immune cell populations within the CT26 tumors by multi-color flow cytometry following 14 d of dosing (see Figure S10 for gating strategy). We identified an increase in tumor-associated CD8+ cells following trametinib treatment that was reversed by co-treatment with JQ1 (Fig. 7e). Trametinib alone and the combination of JQ1 and trametinib also caused a significant increase in CD4+ T cells (Fig. 7e). Notably, the number of Tregs was increased by trametinib treatment and the combination of JQ1 and trametinib (Fig. 7e). JQ1 treatment alone and when combined with trametinib resulted in increased PD-1 expression on CD8+ cells, indicative of T-cell exhaustion (Fig. 7f).

Hypothesizing that increased expression of inflammatory genes may associate with more aggressive disease in the clinic, we identified a panel of 66 genes that associated with MEKi resistance in the CCLE dataset and were suppressed by JQ1 treatment in T84 cells (Table S2). CRC patients with amplification or increased mRNA expression of these genes exhibited a significantly reduced overall survival in the TCGA/cBioPortal dataset [33, 34] (Fig. 7g).

## Discussion

Intrinsic and acquired drug resistance are significant hurdles to overcome to maximize the utility of precision medicine. Our study focussed on understanding why the MEKi trametinib, despite showing good clinical activity in *BRAF*-mutant melanoma, failed to show any clinical response in *KRAS*-mutant CRC [16]. Our data describe a transcriptional state associated with resistance to selective MEKis, defined by interferon and inflammation-mediated responses and involving NFκB activation, constitutively activated in a high proportion of CRCs [35]. To our knowledge, this is the first report to implicate an interferon/inflammatory transcriptional signature in intrinsic or acquired resistance to MEK inhibition. Given the highly inflammatory, cytokine-rich, environment of the colon, as observed in colitis-associated cancer and in heavily pre-treated cancer patients, we propose that inflammation may have rendered tumors resistant to trametinib [16, 36, 37].

JQ1 in combination with trametinib synergistically inhibits the proliferation of MEKi-resistant cell lines and induces apoptosis. Transcriptional profiling implicates the expression of inflammatory genes in MEKi resistance, both abrogated by JQ1. Notably, MEK inhibition was recently shown to overcome resistance to BRD4 inhibition in CRC through suppression of MYC [38]. In our RNA-seq analysis of the *KRAS*-mutant, MEKi-resistant T84 cell line, a *MYC* gene signature suppressed by JQ1 treatment was ranked 13th, with 7 of the top 12 gene sets representing signatures of TNF, interferon and other cytokine-mediated gene expression. However, enrichment of *MYC* gene expression signatures was not observed in our model of acquired resistance to trametinib. Nevertheless, knockdown of MYC by siRNA did sensitize cells to MEK inhibition so is likely to contribute to the antiproliferative effects observed. Overall, our data link interferon and inflammatory gene expression to both mechanisms of intrinsic and acquired resistance to MEK inhibition.

Importantly, we provide evidence that the combination of trametinib and JQ1 is efficacious in PDOs and in vivo using models that display resistance to trametinib. Notably, the PDO cultures did express relatively high levels of cytokines and ISGs that we have implicated in resistance to trametinib. This suggests they are reflective of a more inflammatory state, possibly a consequence of tumor-induced inflammation or in response to prior chemotherapies. Despite the observed antiproliferative activity of JQ1 toward normal colon epithelial cells in the colony formation assays, our in vivo studies demonstrate that the combination of JQ1 and trametinib was tolerated by the mice. However, this does raise concerns that chronic dosing of JQ1 could have undesirable gastrointestinal toxicities in patients that could limit the therapeutic window of this approach. Nevertheless, recent clinical studies have also demonstrated that the first-in-class bromodomain inhibitor birabresib is tolerated by cancer patients with manageable toxicities [39, 40]. The adoption of intermittent dosing strategies may have the potential to limit such effects and emerging bromodomain inhibitors with differing selectivity profiles could conceivably exhibit different toxicity profiles than birabresib. Long-term dosing would likely be required as we have shown that withdrawal of treatment does eventually lead to regrowth of the tumor. Nevertheless, the combination group maintained a significant inhibition of tumor growth relative to the vehicle control out to at least 42 d, which was not observed with the single-agent groups. The immunocompetent CT26 syngeneic model enabled assessment of the immune cell population within the tumor. The increase in tumor-associated CD4+ cells following trametinib treatment, raises the possibility that Th1-polarized CD4+ T cells may contribute to the antitumor activity observed [41]. However, as antitumor activity is only observed in combination with JQ1, an increase in CD4+ cells alone is insufficient to drive efficacy. The combination treatment also significantly increased the number of Tregs, possibly suggestive of an immuno-suppressive mechanism. Finally, increased PD-1 expression on CD8+ cells induced by JQ1, which was further increased when in combination with trametinib, indicates higher levels of T-cell activity and T-cell exhaustion. We speculate this could be due to increased antigen release from dying tumor cells or as yet undiscovered direct effects on immune cells. Overall, given that synergy between trametinib and JQ1 is observed in vitro and in the NCr nude mouse model, where the immune system is either absent or substantially impaired, together with the suppressive effects of JQ1 on immune cell infiltrates in the CT26 syngeneic model, it is likely that synergy arises mainly through direct effects on the tumor cells.

Our association of a 66-gene signature with poor survival in CRC patients is suggestive of the potential clinical relevance of this study and supports the investigation of combinatorial strategies to counter the intrinsic resistance to MEKis observed in CRC [16]. Inflammatory cytokines such as IL1β, CXCL1, and CXCL8 (IL-8) have been linked to cetuximab resistance in CRC [42]. Combining MEKis with clinical stage antagonists of cytokine receptors such as anakinra, which blocks the IL1 receptor, infliximab, which binds TNFα and bermekimab (MABp1), which binds IL1α may yield novel therapeutic strategies to suppress cytokine-mediated resistance [43–45]. However, targeting individual components may conceivably be inferior to a broader blockade. Thus, bromodomain inhibitors may overcome multiple mechanisms of resistance to targeted therapy. Recently, bromodomain inhibition has been shown to suppress enhancer remodeling induced by trametinib and overcome resistance in breast cancer [46]. Moreover, JQ1 treatment has shown synergistic activity with trametinib in malignant peripheral nerve sheath tumors (MPNSTs) driven by neurofibromin 1 (NF1) mutation and polycomb repressive complex 2 (PRC2) loss [47], suggesting further utility of this therapeutic approach in other tumor types. Our data support the continued development of bromodomain inhibitors and further investigation of their utility in combinatorial therapeutic strategies for *KRAS*-mutant CRC, to maximize response to targeted agents and suppress mechanisms of intrinsic and acquired resistance.

## Materials and methods

### Cell culture and reagents

Human and mouse cancer cell lines were obtained from the American Type Culture Collection (Teddington, UK) or the Deutsche Sammlung von Mikroorganismen und Zellkulturen (Braunschweig, Germany) and grown in the recommended culture medium, supplemented with 10% fetal bovine serum, at 37 °C and an atmosphere of 5% CO_2_. Cell lines were routinely tested for mycoplasma and not cultured for longer than 20 passages. PDOs and their culture conditions have been previously described [32]. *KRAS* mutations in PDOs and matching parental tissue were confirmed by targeted next-generation sequencing [32]. Inhibitors were purchased from Stratech Scientific Ltd (Ely, UK). Recombinant cytokines were purchased from Peprotech (London, UK).

### Immunoblotting

Cell lysis and immunoblotting techniques were as previously described [48, 49]. The antibodies used against specific proteins and their concentrations for immunoblotting in this study are listed in the Table S3.

### Cell proliferation assays

Cell proliferation assays were as previously described and quantified using CellTiter-Blue (Promega, Southampton, UK) [49]. The drug response assay used for PDOs has been described in detail [32]. Colony formation assays were conducted as previously described [49]. Where cell counting was used to assay cell proliferation, cells were seeded into six-well plates in triplicate per condition and treated with compounds for 72 h. Viable cell number was determined by Trypan blue staining and normalized to the cell number prior to treatment. For three-dimensional spheroid culture, cells were seeded into 96-well ultra-low attachment plates (Corning, Amsterdam, The Netherlands) and allowed to establish for 48 h prior to treatment. Spheroid diameter was measured over time using imaging cytometry (Celigo, Nexcelom, Manchester, UK).

### Apoptosis assay

Cells were treated with either DMSO or the indicated compounds. After 72 h, cells were stained with annexin V and propidium iodide using the Annexin V Apoptosis Detection Kit (eBioscience, Renfrew, UK) and analyzed by flow cytometry (LSRII, BD Biosciences, Wokingham, UK).

### Quantitative real-time PCR

Total RNA was extracted from cells using the miRNeasy Mini Kit (Qiagen, Manchester, UK) and reverse transcribed using the high capacity complementary DNA reverse-transcription kit (Applied Biosystems, Renfrew, UK). The PCR was performed using the Fast SYBR Green Master Mix (Applied Biosystems) on a ViiA 7 Real-Time PCR System (Applied Biosystems). Primer combinations were designed using the Harvard Primer Bank (http://pga.mgh.harvard.edu/primerbank) (Table S4).

### RNA-sequencing

Total RNA was isolated using the miRNeasy kit (Qiagen, Manchester, UK). RNA samples were quality controlled and sequenced by the Tumor Profiling Unit of the Institute of Cancer Research (ICR, London). NEB (Hitchin, UK) polyA kit was used to select the mRNA. Strand-specific libraries were generated from the mRNA using the NEB ultra directional kit. Illumina paired-end libraries were sequenced on a HiSeq2500 (Illumina, Little Chesterford, UK) using v4 chemistry acquiring 2 × 100 bp reads. Bcl2fastq software (v1.8.4, Illumina) was used for converting the raw base calls to fastqs and to further demultiplex the sequencing data. The paired-end fastq files were used for further analysis. Tophat2 spliced alignment software was used to align reads to the reference genome (GRCH37) in combination with Bowtie2. Once the reads were aligned, HTSeq-count was used to count the number of reads mapping unambiguously to genomic features in each sample. Differential expression analysis of the count data was done in R using the DESeq2 Bioconductor package. The lists of up- and downregulated differentially expressed genes were then tested for enrichment of gene sets uniquely defining the previously defined CRC subtypes [26] using the Gage Bioconductor package [50]. RNA-seq data were deposited at the Gene Expression Omnibus database: GSE118490 for the parental HCT116 cells and HCT116_R4 clone and GSE118548 for the T84 trametinib/JQ1 combination experiment.

### siRNA assays

siRNAs targeting *BRD4* (L-004937-00-0005), *NFkB p65* (L-003533-00-0005), *IFIT1* (L-019616-00-0005), and *MYC* (L-003282-02-005) (ON-TARGET plus SMARTpool, Dharmacon, Cambridge, UK) and a non-targeting siNT-control (D-001810-01-05) were pre-incubated with Lipofectamine RNAiMAX (Thermo Fisher, Renfrew, UK) and Opti-MEM culture medium (Gibco, Renfrew, UK) according to the manufacturer’s instructions. Cells were reverse transfected with the siRNA-lipid complexes and incubated at 37 °C for the indicated time points until further analysis.

### In vivo studies

T84 tumors were established by subcutaneous injection of 5 × 10^6^ cells into the right flank of female NCr mice and randomly allocated into treatment groups. Treatment using published, efficacious schedules was initiated when tumors reached a mean diameter of ~6 mm (indicated as day 0). Control mice (*n* = 10) received vehicle (1% Hydroxypropylbetacyclodextrin (2-hydroxypropyl)-β-cyclodextrin) po, 10% DMSO in 10% w/v Hydroxypropylbetacyclodextrin ip), and treated mice (*n* = 10) were given 1 mg/kg trametinib orally or 50 mg/kg JQ1 administrated by intraperitoneal injection or the combination of both drugs daily for of 28 d [51, 52]. Tumor volumes, using formula 4.91 × (1st diameter/4 + 2nd diameter/4)^3^, and body weights were determined three times weekly. A dosing holiday was given to all groups on day 21 to aid tolerability. CT26 tumors were established by injection of 5 × 10^5^ cells into the right flank of female BALB/C mice and treated as above. All animal studies were approved by the local research ethics committee and carried out in accordance with the UK Animals (Scientific Procedures) Act 1986 and National Guidelines [53]. Appropriate group sizes were determined by power analyses (G*Power ver 3.1.5) and are guided by extensive experience in running such studies. No blinding of groups was done.

### Tumor dissociation

Tumors were dissociated into a single-cell suspension using a gentleMACS Octo Dissociator with Heaters (Miltenyi Biotec, Bisley, UK) and the Mouse Tumor Dissociation Kit (Miltenyi Biotec). Samples were run on the 37C_m_TDK_1 program, applied to a 70 μm MACS SmartStrainer and washed in phosphate-buffered saline (PBS). Erythrocytes were removed from samples by suspension in red blood cell lysis buffer for 5 min at room temperature. Samples were resuspended in PBS for flow cytometry staining.

### Flow cytometry

Cells were stained with a fixable viability dye (Thermo Fisher Scientific) and blocked with an anti-mouse CD16/CD32 antibody (Thermo Fisher Scientific). A panel of fluorescence-conjugated antibodies was added to cell suspensions at specified dilutions (Table S5) and incubated at 4 °C for 30 min. Intracellular staining was performed using the Foxp3/Transcription factor staining buffer set (Thermo Fisher Scientific). Cells were fixed in 4% paraformaldehyde solution. Finally, cells were resuspended in protein blocking agent (PBA), counting beads were added and samples were analyzed on a BD LSR II flow cytometer. Data analysis was performed using FlowJo software (Tree Star Inc., Ashland, Oregon, USA). Gating strategies are shown in Figure S9. Absolute cell counts were calculated as follows: absolute count (cells/µL) = (cell count × counting bead volume)/(counting bead count × cell volume) × counting bead concentration. Cell counts were normalized by dividing the cell count obtained using counting beads by tumor volumes.

## Supplementary information

Supplementary material

Supplementary tables

## References

[1] Wood LD, Parsons DW, Jones S, Lin J, Sjoblom T, Leary RJ (2007). The genomic landscapes of human breast and colorectal cancers. Science.

[2] Cancer Genome Atlas N. (2012). Comprehensive molecular characterization of human colon and rectal cancer. Nature.

[3] Boutin AT, Liao WT, Wang M, Hwang SS, Karpinets TV, Cheung H (2017). Oncogenic Kras drives invasion and maintains metastases in colorectal cancer. Genes Dev.

[4] Golay HG, Barbie DA (2014). Targeting cytokine networks in KRAS-driven tumorigenesis. Expert Rev Anticancer Ther.

[5] Ancrile BB, O’Hayer KM, Counter CM (2008). Oncogenic ras-induced expression of cytokines: a new target of anti-cancer therapeutics. Mol Interv.

[6] Sparmann A, Bar-Sagi D (2004). Ras-induced interleukin-8 expression plays a critical role in tumor growth and angiogenesis. Cancer Cell.

[7] Wislez M, Fujimoto N, Izzo JG, Hanna AE, Cody DD, Langley RR (2006). High expression of ligands for chemokine receptor CXCR2 in alveolar epithelial neoplasia induced by oncogenic kras. Cancer Res.

[8] Jamieson T, Clarke M, Steele CW, Samuel MS, Neumann J, Jung A (2012). Inhibition of CXCR2 profoundly suppresses inflammation-driven and spontaneous tumorigenesis. J Clin Invest.

[9] Ancrile B, Lim KH, Counter CM (2007). Oncogenic Ras-induced secretion of IL6 is required for tumorigenesis. Genes Dev.

[10] Grothey A, Cutsem EV, Sobrero A, Siena S, Falcone A, Ychou M (2013). Regorafenib monotherapy for previously treated metastatic colorectal cancer (CORRECT): an international, multicentre, randomised, placebo-controlled, phase 3 trial. Lancet.

[11] Hurwitz H, Fehrenbacher L, Novotny W, Cartwright T, Hainsworth J, Heim W (2004). Bevacizumab plus irinotecan, fluorouracil, and leucovorin for metastatic colorectal cancer. N Engl J Med.

[12] Van Cutsem E, Kohne CH, Lang I, Folprecht G, Nowacki MP, Cascinu S (2011). Cetuximab plus irinotecan, fluorouracil, and leucovorin as first-line treatment for metastatic colorectal cancer: updated analysis of overall survival according to tumor KRAS and BRAF mutation status. J Clin Oncol.

[13] Van Cutsem E, Peeters M, Siena S, Humblet Y, Hendlisz A, Neyns B (2007). Open-label phase III trial of panitumumab plus best supportive care compared with best supportive care alone in patients with chemotherapy-refractory metastatic colorectal cancer. J Clin Oncol.

[14] Samatar AA, Poulikakos PI (2014). Targeting RAS-ERK signalling in cancer: promises and challenges. Nat Rev Drug Discov.

[15] Flaherty KT, Robert C, Hersey P, Nathan P, Garbe C, Milhem M (2012). Improved survival with MEK inhibition in BRAF-mutated melanoma. N Engl J Med.

[16] Infante JR, Fecher LA, Falchook GS, Nallapareddy S, Gordon MS, Becerra C (2012). Safety, pharmacokinetic, pharmacodynamic, and efficacy data for the oral MEK inhibitor trametinib: a phase 1 dose-escalation trial. Lancet Oncol.

[17] Emery CM, Vijayendran KG, Zipser MC, Sawyer AM, Niu L, Kim JJ (2009). MEK1 mutations confer resistance to MEK and B-RAF inhibition. Proc Natl Acad Sci USA.

[18] Manchado E, Weissmueller S, Morris JP, Chen C-C, Wullenkord R, Lujambio A (2016). A combinatorial strategy for treating KRAS-mutant lung cancer. Nature.

[19] Sun C, Hobor S, Bertotti A, Zecchin D, Huang S, Galimi F (2014). Intrinsic resistance to MEK inhibition in KRAS mutant lung and colon cancer through transcriptional induction of ERBB3. Cell Rep.

[20] Lito P, Saborowski A, Yue J, Solomon M, Joseph E, Gadal S (2014). Disruption of CRAF-mediated MEK activation is required for effective MEK inhibition in KRAS mutant tumors. Cancer Cell.

[21] Barretina J, Caponigro G, Stransky N, Venkatesan K, Margolin AA, Kim S (2012). The Cancer Cell Line Encyclopedia enables predictive modelling of anticancer drug sensitivity. Nature.

[22] Grivennikov SI, Greten FR, Karin M (2010). Immunity, inflammation, and cancer. Cell.

[23] Clevers H (2004). At the crossroads of inflammation and cancer. Cell.

[24] Crusz SM, Balkwill FR (2015). Inflammation and cancer: advances and new agents. Nat Rev Clin Oncol.

[25] Roumeliotis TI, Williams SP, Goncalves E, Alsinet C, Del Castillo Velasco-Herrera M, Aben N (2017). Genomic determinants of protein abundance variation in colorectal cancer cells. Cell Rep.

[26] Sadanandam A, Lyssiotis CA, Homicsko K, Collisson EA, Gibb WJ, Wullschleger S (2013). A colorectal cancer classification system that associates cellular phenotype and responses to therapy. Nat Med.

[27] Brown JD, Lin CY, Duan Q, Griffin G, Federation AJ, Paranal RM (2014). Nf-kb directs dynamic super enhancer formation in inflammation and atherogenesis. Mol Cell.

[28] Zou Z, Huang B, Wu X, Zhang H, Qi J, Bradner J (2014). Brd4 maintains constitutively active NF-kappaB in cancer cells by binding to acetylated RelA. Oncogene.

[29] Blagg J, Workman P (2017). Choose and use your chemical probe wisely to explore cancer biology. Cancer Cell.

[30] Nicodeme E, Jeffrey KL, Schaefer U, Beinke S, Dewell S, Chung C-W (2010). Suppression of inflammation by a synthetic histone mimic. Nature.

[31] Delmore JE, Issa GC, Lemieux ME, Rahl PB, Shi J, Jacobs HM (2011). BET bromodomain inhibition as a therapeutic strategy to target c-Myc. Cell.

[32] Vlachogiannis G, Hedayat S, Vatsiou A, Jamin Y, Fernandez-Mateos J, Khan K (2018). Patient-derived organoids model treatment response of metastatic gastrointestinal cancers. Science.

[33] Cerami E, Gao J, Dogrusoz U, Gross BE, Sumer SO, Aksoy BA (2012). The cBio cancer genomics portal: an open platform for exploring multidimensional cancer genomics data. Cancer Discov.

[34] Gao J, Aksoy BA, Dogrusoz U, Dresdner G, Gross B, Sumer SO (2013). Integrative analysis of complex cancer genomics and clinical profiles using the cBioPortal. Sci Signal.

[35] Sakamoto K, Maeda S, Hikiba Y, Nakagawa H, Hayakawa Y, Shibata W (2009). Constitutive NF-kappaB activation in colorectal carcinoma plays a key role in angiogenesis, promoting tumor growth. Clin Cancer Res.

[36] Romano M, DEF F, Zarantonello L, Ruffolo C, Ferraro GA, Zanus G (2016). From inflammation to cancer in inflammatory bowel disease: molecular perspectives. Anticancer Res.

[37] Terzić J, Grivennikov S, Karin E, Karin M (2010). Inflammation and colon cancer. Gastroenterology.

[38] Ma Y, Wang L, Neitzel LR, Loganathan SN, Tang N, Qin L (2017). The MAPK pathway regulates intrinsic resistance to BET inhibitors in colorectal cancer. Clin Cancer Res.

[39] Berthon C, Raffoux E, Thomas X, Vey N, Gomez-Roca C, Yee K (2016). Bromodomain inhibitor OTX015 in patients with acute leukaemia: a dose-escalation, phase 1 study. Lancet Haematol.

[40] Lewin Jeremy, Soria Jean-Charles, Stathis Anastasios, Delord Jean-Pierre, Peters Solange, Awada Ahmad, Aftimos Philippe G., Bekradda Mohamed, Rezai Keyvan, Zeng Zhen, Hussain Azher, Perez Susan, Siu Lillian L., Massard Christophe (2018). Phase Ib Trial With Birabresib, a Small-Molecule Inhibitor of Bromodomain and Extraterminal Proteins, in Patients With Selected Advanced Solid Tumors. Journal of Clinical Oncology.

[41] Nagarkatti M, Clary SR, Nagarkatti PS (1990). Characterization of tumor-infiltrating CD4+T cells as Th1 cells based on lymphokine secretion and functional properties. J Immunol.

[42] Gelfo V, Rodia MT, Pucci M, Dall’Ora M, Santi S, Solmi R (2016). A module of inflammatory cytokines defines resistance of colorectal cancer to EGFR inhibitors. Oncotarget.

[43] Hickish T, Andre T, Wyrwicz L, Saunders M, Sarosiek T, Kocsis J (2017). MABp1 as a novel antibody treatment for advanced colorectal cancer: a randomised, double-blind, placebo-controlled, phase 3 study. Lancet Oncol.

[44] Knight DM, Trinh H, Le J, Siegel S, Shealy D, McDonough M (1993). Construction and initial characterization of a mouse-human chimeric anti-TNF antibody. Mol Immunol.

[45] Shouval DS, Biswas A, Kang YH, Griffith AE, Konnikova L, Mascanfroni ID (2016). Interleukin 1beta mediates intestinal inflammation in mice and patients with interleukin 10 receptor deficiency. Gastroenterology.

[46] Zawistowski JS, Bevill SM, Goulet DR, Stuhlmiller TJ, Beltran AS, Olivares-Quintero JF (2017). Enhancer remodeling during adaptive bypass to MEK inhibition is attenuated by pharmacologic targeting of the P-TEFb complex. Cancer Discov.

[47] De Raedt T, Beert E, Pasmant E, Luscan A, Brems H, Ortonne N (2014). PRC2 loss amplifies Ras-driven transcription and confers sensitivity to BRD4-based therapies. Nature.

[48] Whittaker SR, Walton MI, Garrett MD, Workman P (2004). The Cyclin-dependent kinase inhibitor CYC202 (R-roscovitine) inhibits retinoblastoma protein phosphorylation, causes loss of Cyclin D1, and activates the mitogen-activated protein kinase pathway. Cancer Res.

[49] Whittaker SR, Theurillat JP, Van Allen E, Wagle N, Hsiao J, Cowley GS (2013). A genome-scale RNA interference screen implicates NF1 loss in resistance to RAF inhibition. Cancer Discov.

[50] Luo W, Friedman MS, Shedden K, Hankenson KD, Woolf PJ (2009). GAGE: generally applicable gene set enrichment for pathway analysis. BMC Bioinform.

[51] Filippakopoulos P, Qi J, Picaud S, Shen Y, Smith WB, Fedorov O (2010). Selective inhibition of BET bromodomains. Nature.

[52] Gilmartin AG, Bleam MR, Groy A, Moss KG, Minthorn EA, Kulkarni SG (2011). GSK1120212 (JTP-74057) is an inhibitor of MEK activity and activation with favorable pharmacokinetic properties for sustained in vivo pathway inhibition. Clin Cancer Res.

[53] Workman P, Aboagye EO, Balkwill F, Balmain A, Bruder G, Chaplin DJ (2010). Guidelines for the welfare and use of animals in cancer research. Br J Cancer.

