# Correction: Suppression of interferon gene expression overcomes resistance to MEK inhibition in *KRAS*-mutant colorectal cancer

**DOI:** 10.1038/s41388-019-0835-1

**Published:** 2019-05-30

**Authors:** Steve Wagner, Georgios Vlachogiannis, Alexis De Haven Brandon, Melanie Valenti, Gary Box, Liam Jenkins, Caterina Mancusi, Annette Self, Floriana Manodoro, Ioannis Assiotis, Penny Robinson, Ritika Chauhan, Alistair G. Rust, Nik Matthews, Kate Eason, Khurum Khan, Naureen Starling, David Cunningham, Anguraj Sadanandam, Clare M. Isacke, Vladimir Kirkin, Nicola Valeri, Steven R. Whittaker

**Affiliations:** 1 Division of Cancer Therapeutics, The Institute of Cancer Research, London, UK; 2 Division of Molecular Pathology, The Institute of Cancer Research, London, UK; 3 Breast Cancer Now Research Centre, The Institute of Cancer Research, London, UK; 4 Tumour Profiling Unit, The Institute of Cancer Research, London, UK; 5 Department of Medicine, Royal Marsden NHS Foundation Trust, London, UK

**Keywords:** Colorectal cancer, Cancer genomics

**Correction to: Oncogene**

10.1038/s41388-018-0554-z, published online 23 October 2018

Following publication of this article it was noted that Supplementary Tables 1–5 were omitted.

These have now been added to the HTML version of the Article.

