# Supplementary material for: Suppression of interferon gene expression overcomes resistance to MEK inhibition in KRAS-mutant colorectal cancer
Source: Oncogene. 2018 Oct 23;38(10):1717–33. doi: 10.1038/s41388-018-0554-z (PMC6462854; doi:10.1038/s41388-018-0554-z)
Supplement: Supplementary file 1 — Supplementary material [file 41388_2018_554_MOESM1_ESM.pdf]

Supplementary Data

Suppression of interferon gene expression overcomes resistance to MEK inhibition in *KRAS*-mutant colorectal cancer

Wagner et al.

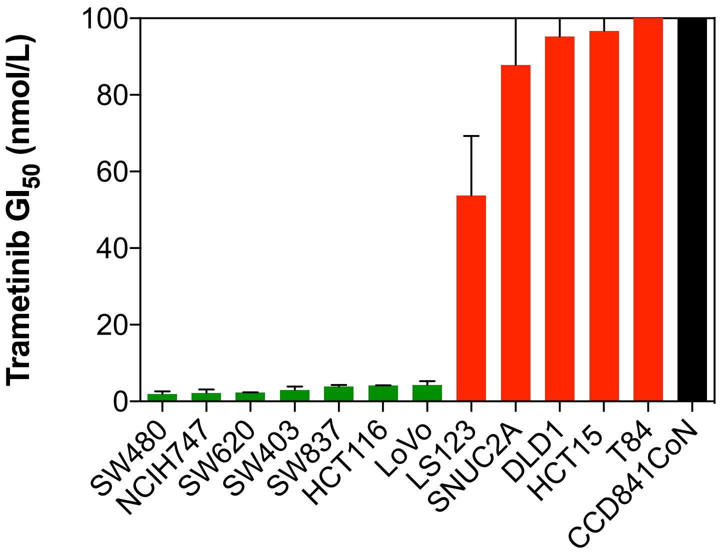

Figure S1. MEK-inhibitor resistant cell lines exhibit reactivation of the MAPK pathway following treatment with trametinib.

Sensitivity to the MEK inhibitor trametinib was assessed in *KRAS*-mutant colorectal cancer cell lines and the normal epithelial colon cell line CCD841CoN exposed to increasing concentrations of the compound for 4 d. Cell number was measured by the CellTiter-Blue assay and a GI_{50} was calculated for each cell line. Average GI_{50} values are plotted; error bars represent standard error (n=3).

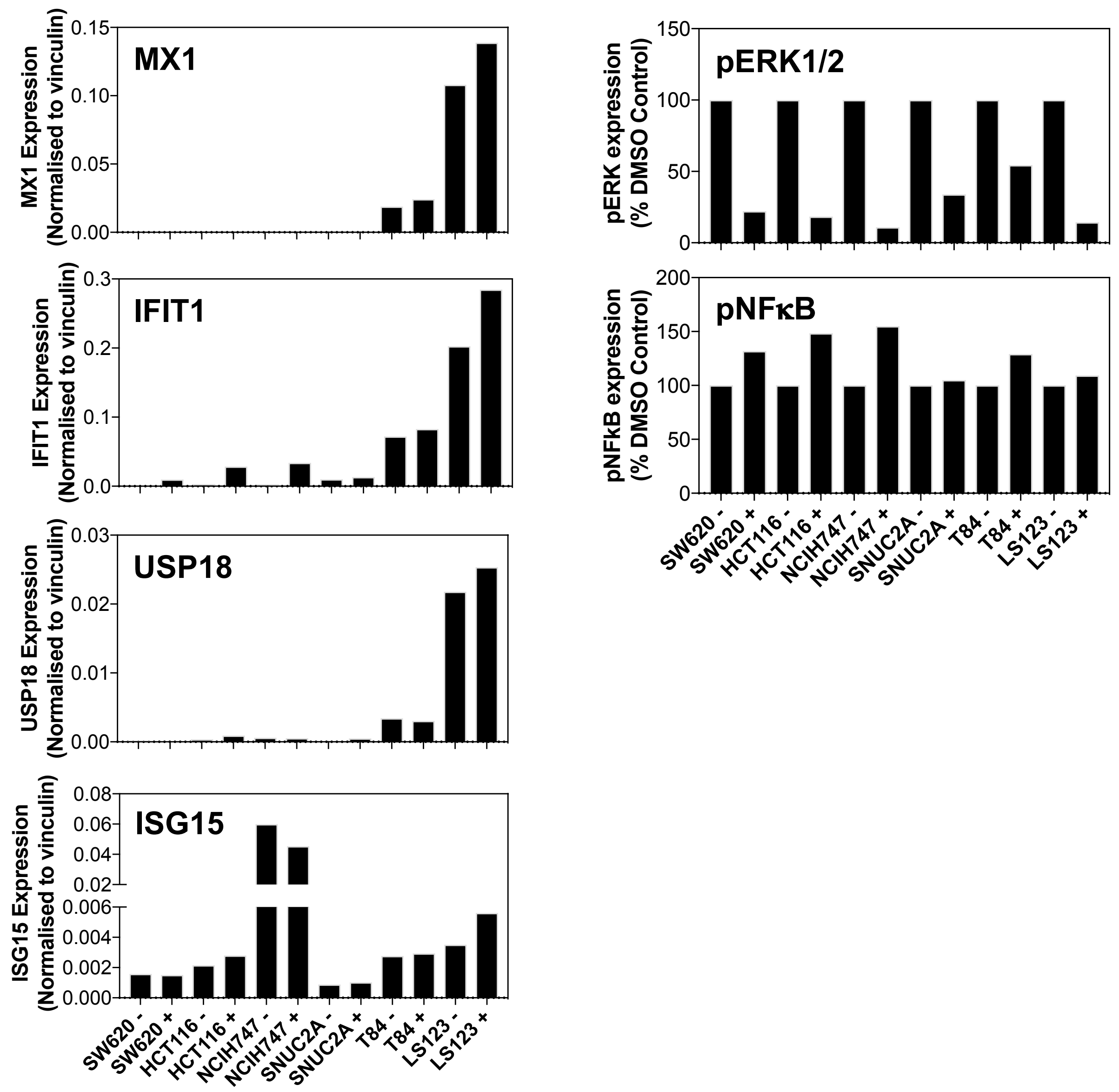

Figure S2. Quantification of Western blots from Figure 1E.

Cells were treated and analysed as described in Figure 1E. DMSO treatment is indicated by '–' and trametinib treatment is indicated by '+'. Western blot images were quantified using the Li-Cor Image Studio software and normalised to vinculin. For phospho-proteins, these were firstly normalised to vinculin (both phospho- and total-protein), then these normalised values were used to express the phospho-protein relative to the total-protein and was then expressed as a percentage of the vehicle control.

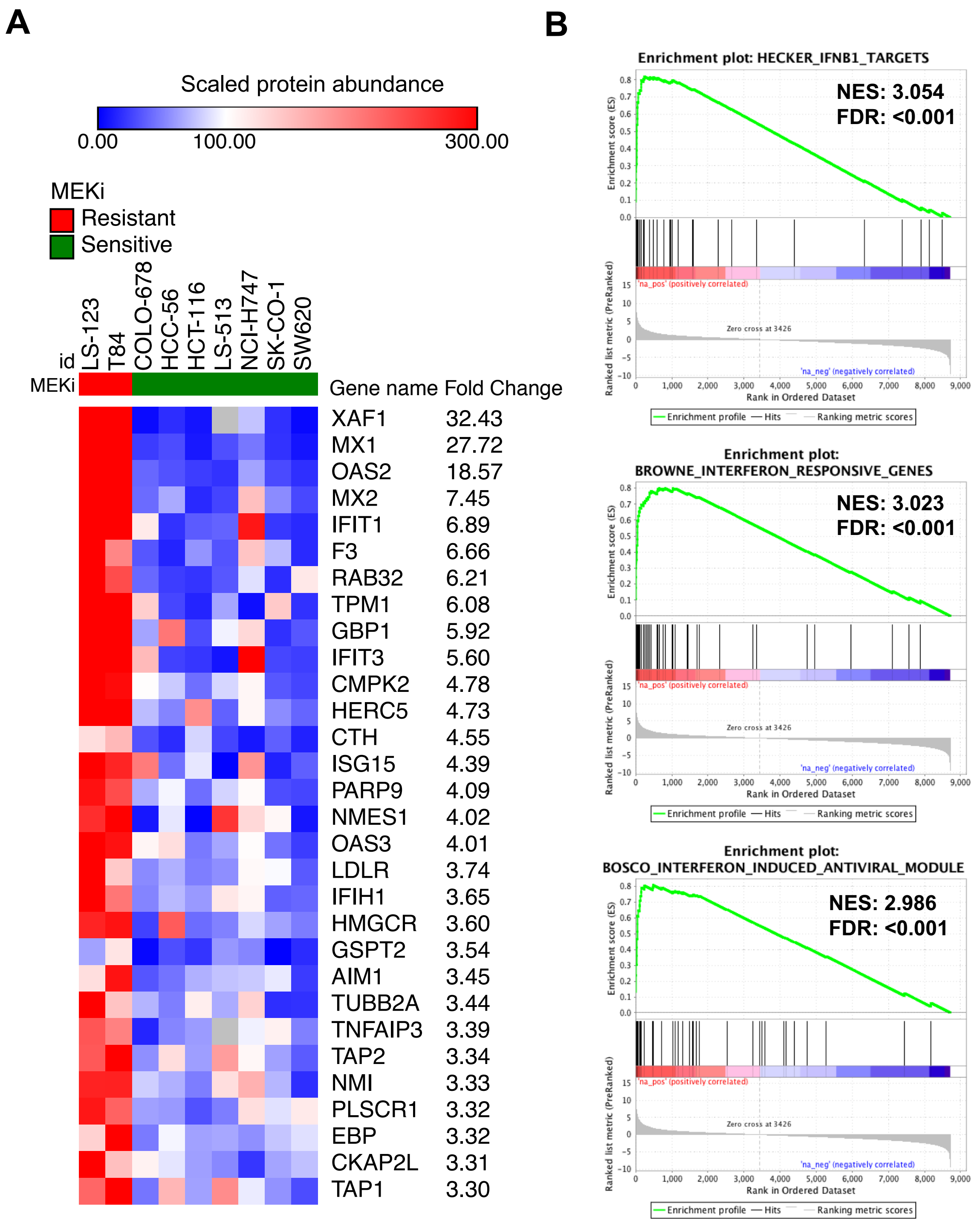

Figure S3. Interferon stimulated genes show increased protein expression in trametinib-resistant cell lines.

A. Comparative marker selection (T-test) of basal protein expression, as determined by isobaric labelling and tribrid mass spectrometry, identified increased expression of ISGs in trametinib (MEKi) resistant cell lines T84 and LS123. The top 30-differentially expressed proteins are shown, ordered by fold-change in expression.

B. GSEA analysis of the rank-ordered, differentially expressed proteins, demonstrated an enrichment of interferon gene sets in MEKi resistant cell lines.

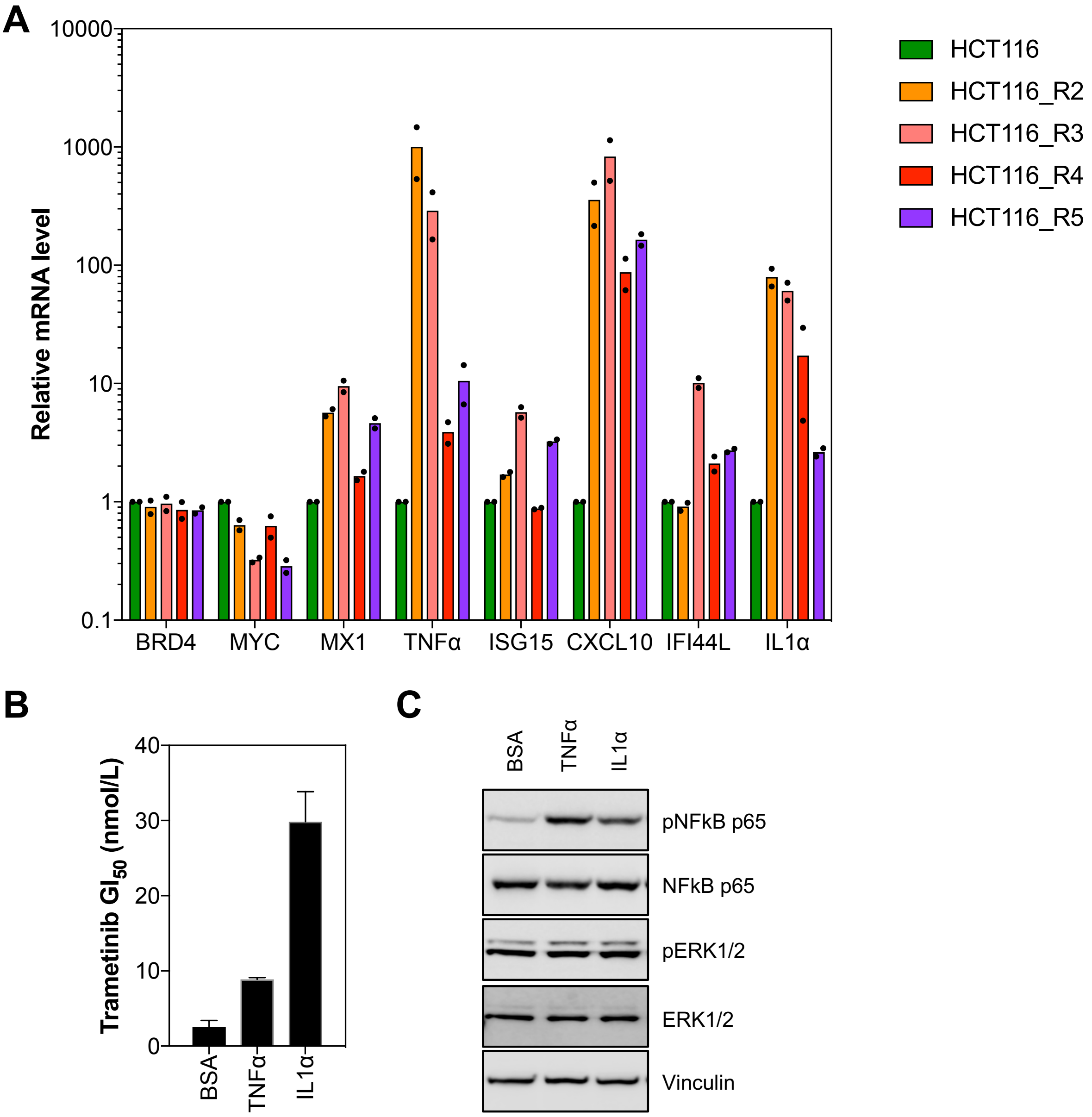

Figure S4. Elevated expression of TNFα and IL1α is associated with acquired resistance to trametinib and recombinant TNFα and IL1α are sufficient to confer resistance to trametinib.

A. HCT116 cells were treated as described in Figure 2A to yield trametinib-resistant clones. mRNA was extracted and analyzed for the expression of the indicated genes, bars represent the mean of two determinations.

B. HCT116 cells were treated with 20 ng/ml BSA, TNFα or IL1α then treated with increasing concentrations of trametinib for 4 d. GI_{50} values were calculated and bars represent the mean GI_{50} value ± SEM (n=4).

C. HCT116 cells were treated with 20 ng/ml BSA, TNFα or IL1α for 16 h and cell lysates analyzed by Western blotting for the indicated proteins.

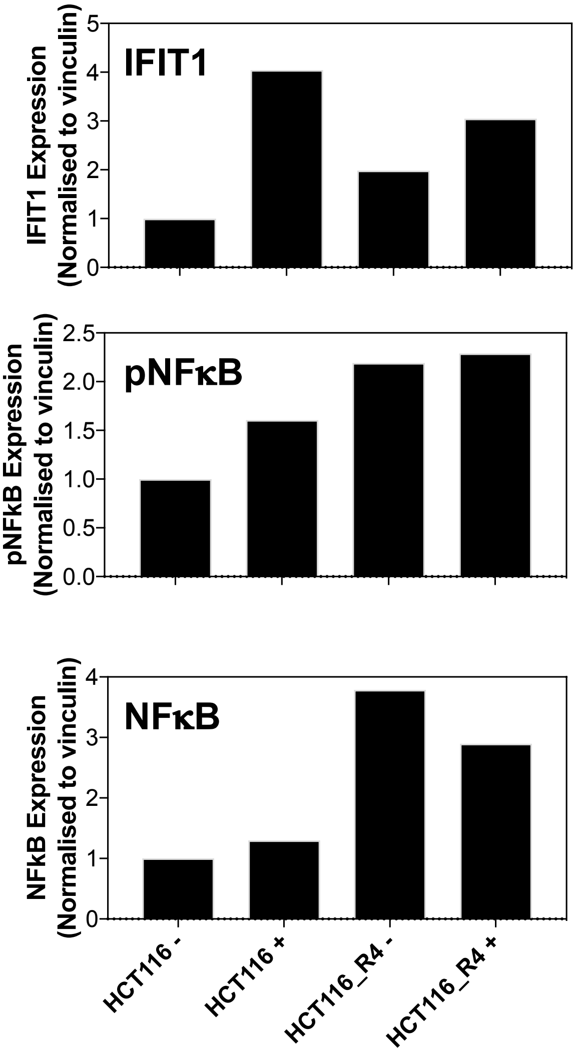

Figure S5. Quantification of Western blots from Figure 2E.

Cells were treated and analysed as described in Figure 2E. DMSO treatment is indicated by '–' and trametinib treatment is indicated by '+'. Western blot images were quantified using the Li-Cor Image Studio software and normalised as indicated.

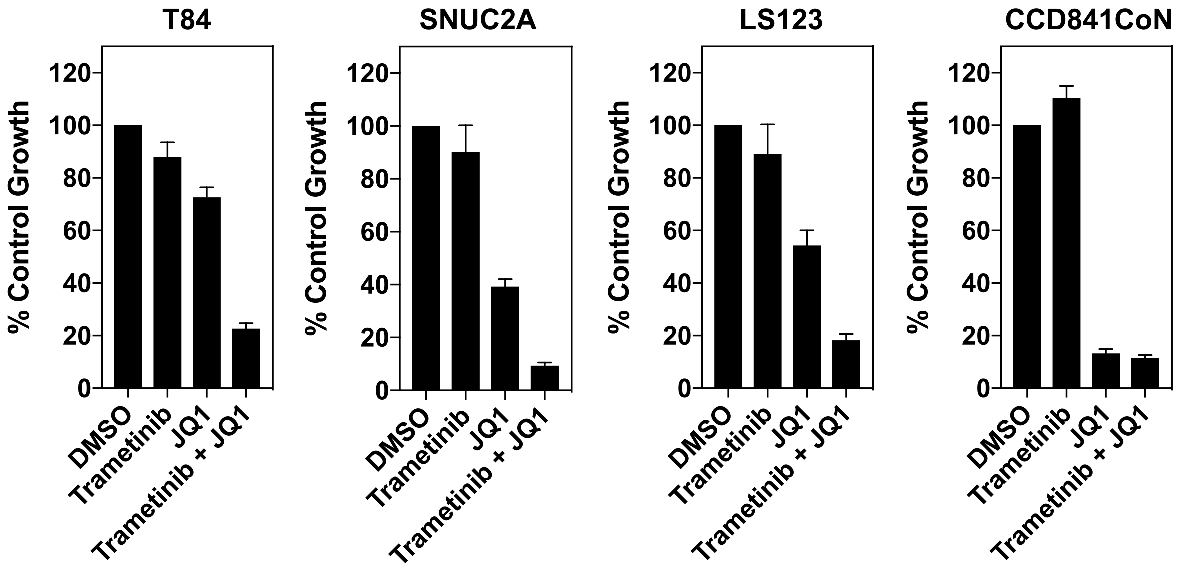

Figure S6. Quantification of colony formation assays in Figure 3D.

T84, SNUC2A, LS123 and CCD841CoN cells were treated as described in Figure 3D. For quantification of relative cell proliferation, crystal violet dye was resuspended in 10% acetic acid and absorbance measured at 595 nm. Values are expressed as a percentage of the control (DMSO) and represent the mean ± SEM (n=3).

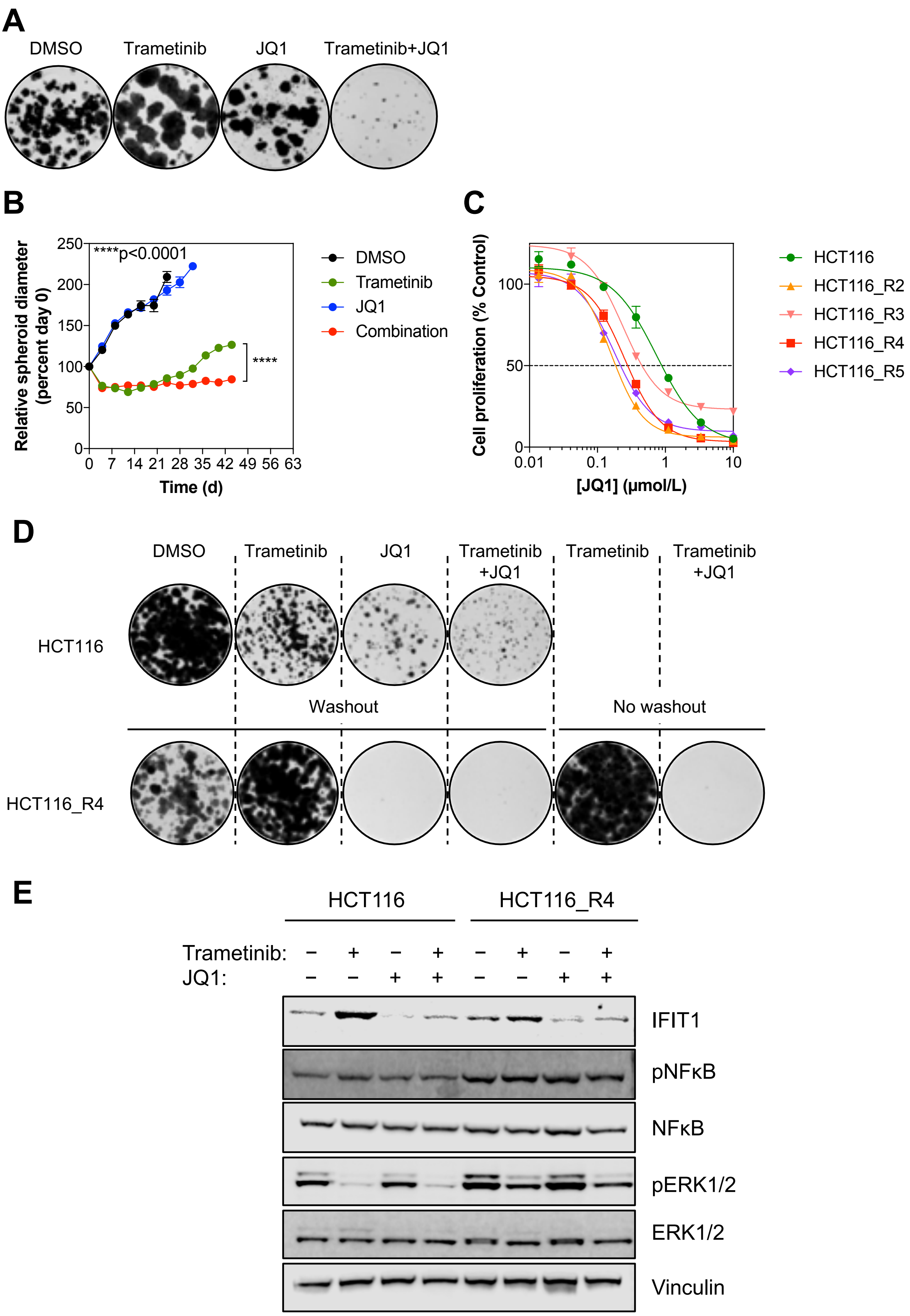

Figure S7. Bromodomain inhibition prevents or reverses the development of acquired resistance to MEK inhibition.

A. HCT116 cells were treated with DMSO, 30 nmol/L trametinib, 0.3 µmol/L JQ1 or their combination for 4 weeks. Cell number was assessed by colony formation assay (n=3).

B. HCT116 cells were cultured as spheroids in suspension and treated with DMSO, 10 nM trametinib, 0.6 µmol/L JQ1, or their combination for the indicated times. Spheroid diameter was measured by Celligo. Results of a single experiment are shown, carried out with 6 replicates per condition. Average spheroid diameter is plotted; error bars represent standard error.

C. HCT116 and MEK-inhibitor resistant subclones R2-R5 were treated with increasing concentrations of JQ1 for 4 d. Cell proliferation was assessed by the CellTiter-Blue assay and GI_{50} values were determined by non-linear regression. Average cell proliferation values shown as a percentage of control cells are plotted; error bars represent standard error.

D. HCT116 and HCT116_R4 cells were treated with 30 nmol/L trametinib, 0.3 µmol/L JQ1, or both compounds in combination for 14 d. HCT116_R4 cells were cultured in the presence of 30 nmol/L trametinib, and this was either washed out prior to JQ1 treatment or left on the cells and JQ1 was added in combination. Cell proliferation was assessed by colony formation assay.

E. HCT116 and HCT116_R4 cells were treated with 30 nmol/L trametinib, 0.3 µmol/L JQ1 or both compounds in combination for 24 h. Cell lysates were analyzed by Western blotting for the indicated proteins.

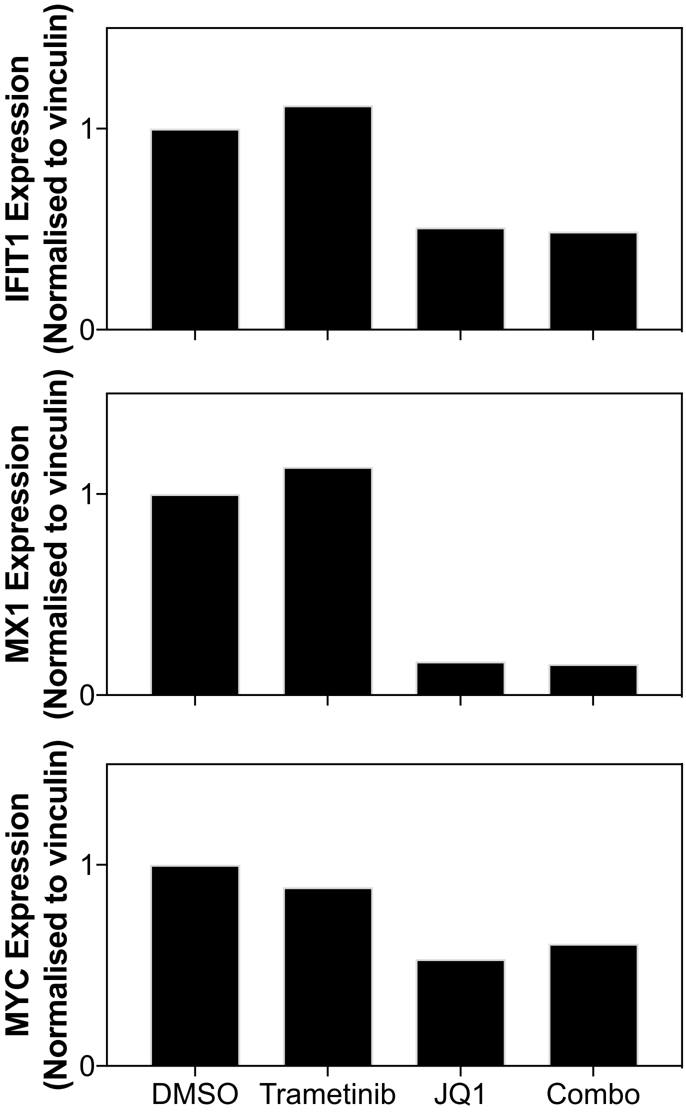

Figure S8. Quantification of Western blots from Figure 6E. Organoid cultures were treated as described in Figure 6E. Western blot images were quantified using the Li-Cor Image Studio software, normalised to a vinculin loading control and expressed relative to the DMSO control.

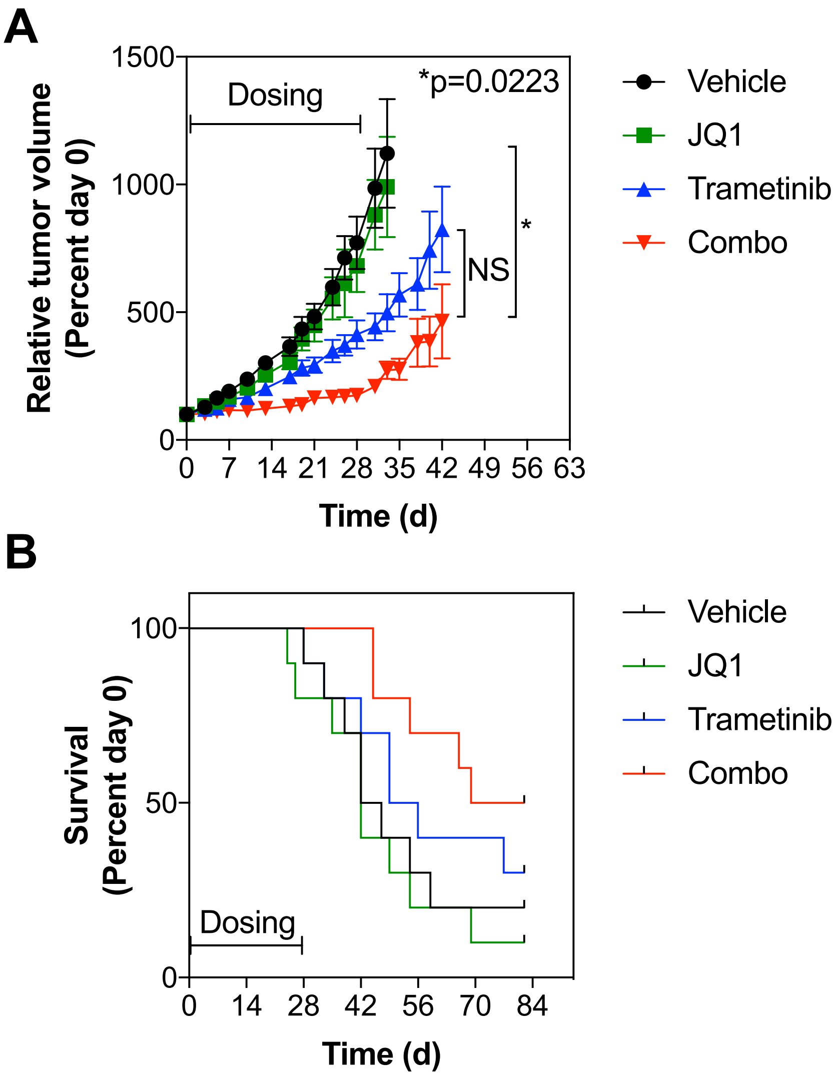

	Vehicle	JQ1	Trametinib	Combo
Vehicle		0.6265	0.3412	0.0512
JQ1			0.1397	0.0131
Trametinib				0.4357
Combo				

Figure S9. The combination of trametinib and JQ1 inhibits the growth of the T84 xenograft model and extends median survival.

A. Human T84 cells (5x10^6/mouse) were inoculated subcutaneously into the flank of NCr mice, n=10 mice per group. Mice were treated with either vehicle, 1 mg/kg/d po trametinib, 50 mg/kg/d ip JQ1, or the combination of trametinib and JQ1 for up to 28 d. Dosing on day 21 was withheld from all groups to aid tolerability. Animals were monitored for tumor regrowth following cessation of treatment. Tumor volume was measured by callipers every 3-5 d, and

the mean volume per group was expressed as a percentage relative to day 0; error bars represent standard error.

B. Survival analysis of the experiment described above and in Figure 7A. Increased median survival was observed with combination treatment versus JQ1 or trametinib alone. Significant differences in survival were calculated by Log-rank (Mantel-Cox) test in a pair-wise fashion and p-values between groups are presented in the table.

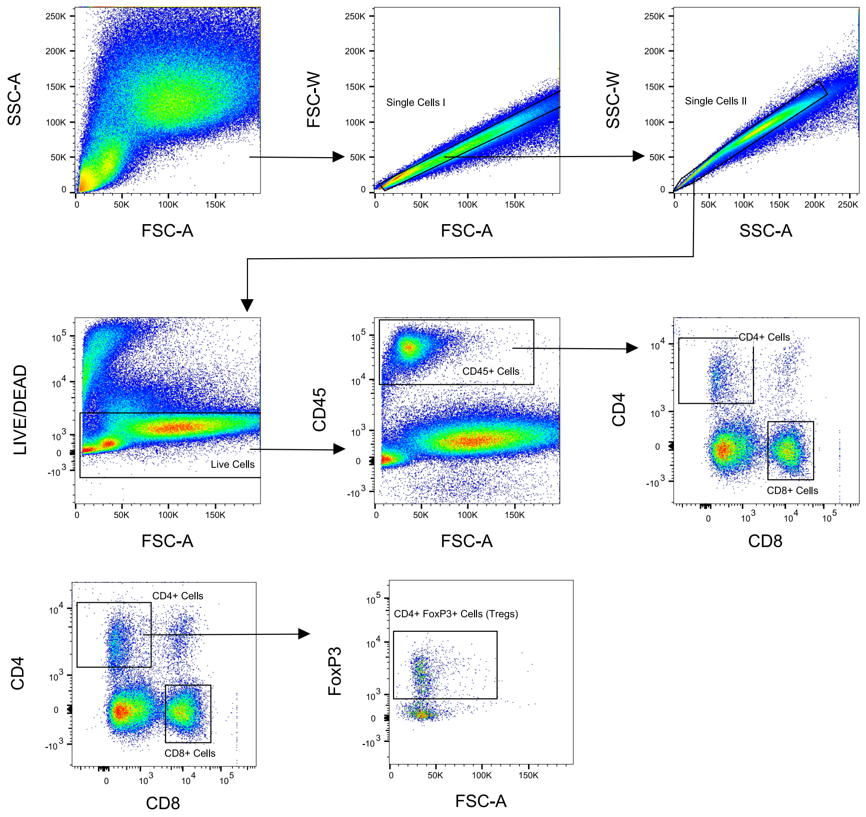

Figure S10. Profiling of immune cell infiltrates in CT26 tumors treated with JQ1 and trametinib.

Gating strategy employed for quantification of T cell populations by flow-cytometry.
